# An oral toxicity assessment of a mosquito larvicidal transgenic algae (*Chlamydomonas reinhardtii*) using adult Zebrafish and its embryos

**DOI:** 10.1371/journal.pone.0303352

**Published:** 2024-06-13

**Authors:** Fareeha Amjad, Hamza Khan, Muhammad Islam Khan, Sidra Ayub, Rashid Bhatti, Rabbia Pervaiz, Kausar Malik, Mohsin Ahmad Khan

**Affiliations:** 1 Nutraceuticals and Microbial Biotechnology Lab, National Center of Excellence in Molecular Biology (CEMB), University of the Punjab, Lahore, Pakistan; 2 Qarshi University, Lahore, Pakistan; Universidade Federal do Para, BRAZIL

## Abstract

Mosquito-borne diseases pose a global health threat, with pathogens like Malaria, Dengue fever, and others transmitted by mosquitoes. Our study focuses on evaluating the toxicity of genetically engineered mosquito larvicidal algae (*Chlamydomonas reinhardtii*) to non-target organisms, specifically Zebrafish. We conducted a 90-day experiment, feeding Zebrafish different combinations of larvicidal algae and commercial fish feed. Statistical analysis revealed no significant differences in mortality, allergenicity, or moribundity among groups. Hematology, molecular analysis, and necropsy showed no physiological differences. Our findings indicate that the transgenic algae (TN72.cry11Ba) had no adverse effects on adult Zebrafish or their larvae. This study confirmed the safety of algae on non-target organisms, such as zebrafish.

## 1. Introduction

In our highly interdependent and globalised society, the potential for the spread of the disease has increased dramatically. Rapid globalisation in the trade and tourism industries, as well as mass migration, have had devastating effects on human existence [1, 2]. Dengue fever and other mosquito-borne illnesses are on the rise because of the cumulative impacts of shifts in the climate and globalization on local weather patterns [3, 4]. Some of the deadliest human illnesses, such as Malaria and Dengue fever, have been spread by mosquitoes and have had devastating effects on human populations throughout history [5]. Many parts of the world are now experiencing the devastating effects of newly emerging mosquito-borne diseases including Zika and Yellow fever [6–9].

A novel strategy involves the selection of specific microorganisms that are eaten by mosquito larvae, followed by genetic engineering to introduce larvicidal genes isolated from Bacillus thuringiensis (Bt) [10]. This approach provides a distinct advantage over conventional B. thuringiensis (Bt) microbial sprays because it eliminates the need for repetitive applications [11, 12]. The traditional spray method requires multiple applications owing to its limited long-term viability, insufficient self-sustainability, susceptibility to degradation by environmental factors, and challenges in delivering the spray to precise larval feeding locations. The use of genetically engineered microorganisms that produce larvicidal genes bypasses these limitations and provides a more efficient and effective approach to control mosquito larvae [13]. Codon optimisation of the cry11Ba gene in the green microalga (*Chlamydomonas reinhardtii*) increased the expression of a protein that killed the mosquito larvae. At a concentration of only 1×10^5^ cells mL-1, this genetically modified alga caused a 100% mortality rate in *Aedes aegypti* [14].

Potential hazards, possible dangers, and unforeseen effects on environmental and human health are raised by the promises and the prospective use of genetically modified algae to feed the world’s rising population [15, 16]. A commonly held belief is that introducing GMOs into the ecosystem can lead to unregulated survival and proliferation, resulting in detrimental consequences [17]. The complexity of ecosystems and the unpredictability of environmental conditions have raised concerns among some academics and concerned government authorities regarding the introduction of GMOs [18, 19]. There have been 40 years of safe use of *Bacillus thuringiensis* (Bt) microbial sprays [20–22], and multiple studies have demonstrated rapid degradation of cry protein following its assimilation into the soil [20]. Bt proteins have no effect on the human or animal gut because there are no appropriate receptors as the gut and intestines of both humans and animals have an alkaline pH [11, 23]. However, risk assessment of transgenic algae for non-target species is lacking. The protein known as cry11Ba was cloned in *C*. *reinhardtii*; however, there has been no previous safety assessment, so it is crucial to conduct a biosafety review of this transgenic algae.

Early biosafety assessments of genetically modified algae can be conducted through substantial equivalency analysis. This involves comparing the molecular characteristics, composition, phenotype, and genotype of GM ingredients with those of their isogenic (non-GM) counterparts. By examining these properties, researchers can determine whether GM algae are substantially equivalent to their non-GM counterparts, thereby providing valuable insights into their biosafety [24]. However, pleiotropic effects cannot be demonstrated using these methods. As a result, laboratory animal testing offers a solution to this dilemma. Zebrafish are often used as model animals in biosafety investigations because of their genetic similarity and common cellular functions [25–27]. It is widely documented that laboratory animals under experimentation cannot be fed solely transgenic feed when investigating the biosafety of transgenic organisms; rather, particular quantities must be generated by mixing the transgenic feed with a conventional diet [28, 29]. The true biosafety evaluation could be jeopardised if animals are fed full feed in the lab, regardless of nutritional effects [30, 31]. Therefore, the GM algal biomass was tested for repeated sub-chronic oral dosage toxicity at three different concentrations i.e 1x10^5^ cells/mL, 2x10^5^ cells/mL, and 5x10^4^ cells/mL mixed with commercially available feed. This was done to ensure that the effect of transgenic algae as algae was self-sustainable. The mosquito larvae were killed when treated with 1 × 10^5^ cells/mL. However, none of these concentrations had any effect on non-target organisms, such as zebrafish, as shown in this study.

The potential risks of GMOs to the health of animals and humans, as well as their environment, are now routinely assessed. Animal feeding tests are required to determine the safety of GM algae for use with non-target species [32]. To determine whether transgenic organisms are safe for non-target organisms, the European Food Safety Authority (EFSA), the Environmental Protection Agency (OPPTS 885.3600), and the Institutional Animal Ethics Committee (IAEC) at CEMB, all proposed to conduct a study to assess the potential oral toxicity over a period of 90 days, with repeated administration of concentrations. The aim of this study was to examine the chronic toxicity of GM algae in non-target organisms, such as insects, rats, and zebrafish, over a course of 90 days, as recommended by the IARC.

## 2. Materials and methods

### 2.1 Bioethics

All procedures involving animals in this investigation were approved by the Institutional Animal Ethics Committee (IAEC) (Ref: IAEC09-2020). Centre of Excellence in Molecular Biology (CEMB) at the University of the Punjab in Lahore, Pakistan, adheres to (IAEC guidelines are in agreement with Guide for the care and use of laboratory animals, National Research Council) the National Research Council’s recommendations for the treatment of animals used in scientific research.

### 2.2 Animal and housing

One hundred and forty zebrafish were obtained from a vendor and placed in a dedicated algal culture room at CEMB. The fish were 7–8 weeks old, with an average weight of 0.256 g at the beginning of the experiment. They were housed in individual tanks (20 fish per tank). The fish were fed twice daily, with a gap of approximately 8–10 hours between feeding times. The optimal conditions for the fish were maintained, including a temperature range of 20–30°C, a light-dark cycle of 12 hours, and a constant supply of oxygen. The study protocols, housing, and treatment methods were approved by the CEMB’s Institute Animal Ethics Committee (IAEC) at the University of Punjab.

### 2.3 Test and control substances

In the Nutraceuticals and Microbial Biotechnology Laboratory of CEMB, PU in Lahore, Pakistan, the cry11Ba gene was cloned into *Chlamydomonas reinhardtii* strain TN72 to produce the mosquito larvicidal Bt protein. In contrast to the isogenic control, TN72 cells transformed with an empty vector pSRSap1 served as the test substance. They were freshly grown every week and orally administered to the zebrafish mixed with commercially available feed. Algae may persist in water for a long time because it is its preferred medium for growth. Finally, this transgenic algae will be applied in aquatic environments as a mosquito larvicide, where off-target animal species can also eat and drink it. Testing the biosafety of aquatic animals, such as zebrafish, is important. An aqueous solution, is recommended by the OECD for administering the test chemical. Murbach et al. [32] and Fields et al. [33], and Yadav et al. [34] have all done oral biosafety investigations of *C*. *reinhardtii* with similar results.

*C*. *reinhardtii* CC-5168 cw15 psbH (TN72 strain) was acquired from the Chlamydomonas Resource Centre, located in the United States of America. This particular strain possesses a deficiency in cell wall structure due to a cw15 nuclear mutation. Additionally, psbH, which is associated with photosystem II, was deliberately removed. Within the excised region, an aadA cassette was inserted, conferring spectinomycin resistance and facilitating spectinomycin resistance selection as a means of targeting this strain. The TN72 strain was genetically modified by introducing the cry11Ba gene located in the pSRsap1 vector, which encodes the cry11Ba protein known for its larvicidal activity against mosquitoes. This transgenic microalga was produced at the Nutraceuticals and Microbial Biotechnology Laboratory of the Center for Excellence in Molecular Biology (CEMB) at Punjab University (PU), Lahore, Pakistan. The cloned strain, namely, the cry11Ba gene (in the pSRsap1 vector), served as the test substance in the experiments. TN72 strain transformed with the empty pSRsap1 vector was used as an isogenic or reference control. They were freshly grown every week and given to the Zebrafish orally mixed with commercially available feed. The transgenic Chlamydomonas (TN72. cry), and the isogenic control (TN72. pSRSap1) were grown in TAP (Tris-Acetate-Phosphate) broth on TAP culture plates. After 6 days of algal growth in broth, cell counting was carried out using a haemocytometer to establish their concentration. The broth was centrifuged at 5000 rpm at 4°C for 5 min to separate the algal cells from the TAP media. The supernatant (TAP broth) was discarded and the pellet (algal cells) was resuspended in water. A haemocytometer was used for cell counting under a compound light microscope. For this, 10 μL of the algal cell culture was added to 85 μL of TAP/PBS solution and 5 μL of iodine tincture. The mixture was then vortexed. Approximately 10 μL of the solution was poured on the haemocytometer, and the cells were counted under a microscope at 100X. After cell counting, the obtained algal cells were concentrated or diluted to maintain different test concentrations of the feed. The concentrations used were 1 × 10^5^ cells/mL, 2 × 10^5^ cells/mL, and 5 × 10^4^ cells/mL.

### 2.4 Composition of diet

60 g commercially available crushed fish feed was taken from a local market. Fresh algae were grown in 1000mL of TAP media. Cell count was performed, and contamination was checked to ensure that no bacterial or fungal growth was present in the algal cultures. From 1000mLvolume of the grown algae, 2 g of the pellet was obtained. The pellets were dried to obtain the required concentrations of feed mixed with transgenic algae. After 24 h, the fully dried algal pellets were obtained. The pellets were crushed to obtain a powder using an autoclaved mortar and pestle. For the experimental groups, the following final concentrations of feed were prepared and given to the zebrafish: 1 × 10^5^ cells/mL, 2 × 10^5^ cells/mL, and 5 × 10^4^ cells/mL. The significance of these concentrations is that × 1x10^5^ cells/mL of algae showed 100% lethality of mosquito larvae. In this study, we used double and half concentrations of algal cells to ensure that they worked at various concentrations. This was done mainly because algae are self-sustainable organisms that can grow at various concentrations. However, in the experimental groups, transgenic algae were used. The GM *C*. *reinhardtii* algal strain TN72 with the cry11Ba gene (TN72. cry) was added to the feed. The conventional group was fed algae with a transformed empty vector pSRSap1 in TN72 without the Bt gene (TN72. pSRSap1). This was administered at the same concentration i.e., 1x10^5^ cells/mL, 2 × 10^5^ cells/mL, and 5 × 10^4^ cells/mL as the transgenic group.

### 2.5 Experimental design and treatment

Zebrafish were divided into seven groups S1 Fig, each with 20 fish, irrespective of sex. The division of zebrafish was random; therefore, the body weight did not differ significantly.

This study targeted three concentrations i.e 1x10^5^ cells/mL, 2 × 10^5^ cells/substance, and 5 × 10^4^ cells/mL. The concentration of 1 × 10^5^ cells/mL resulted in 100% complete mortality of mosquito larvae of *Aedes aegypti* in the initial experiments. Assessing the toxic effects at these concentrations was essential to ensure that the non-target organisms were safe from the transgenic algae, as the algae are self-sustainable and the concentration of growth can vary depending on the conditions. Before conducting a field trial, it was necessary to establish biosafety measures. The guidelines provided by the IAEC do not impose any restrictions on concentration levels. Therefore, appropriate concentrations were selected for toxicity assessment. However, the minimum concentration level specified by the Office of Pesticide Programs (OPPTS) guideline 885.3600, which is 10^8^ cells, would be impractical for implementation. This is because achieving such a concentration level would necessitate a concentration process exceeding a factor of 1000, as well as the production of a very large quantity of algae on a weekly basis.

### 2.6 Clinical observations

Throughout the duration of the experiment, all zebrafish were closely monitored and fed twice daily to ensure the absence of mortality, morbidity, or toxicity. This included meticulous observation of any skin abnormalities or irregular movements. Moreover, a comprehensive physical examination was conducted on a weekly basis to detect the presence of skin lesions, abnormal movements, alterations in behavior, swimming patterns, eye abnormalities, or any other physical irregularities. To conclude the experiment, the functional parameters of the zebrafish were assessed. These parameters encompassed the sensory reactivity towards sound, sight, and body-position-related stimuli, evaluation of grip strength, assessment of physical movement, observation of convulsions, shakiness, and abnormal respiration patterns. Throughout the study period, the zebrafish were consistently fed the experimental and control diets. Individual body weights were recorded at the start of the experiment and before its conclusion.

### 2.7 Hematology

Upon sacrificing the zebrafish, whole blood samples were collected using the centrifugation method following anesthesia administration. Approximately 1 mL of blood was obtained and placed in an anticoagulated tube containing ethylenediaminetetraacetic acid (EDTA) to prevent coagulation. These samples were then dispatched to the University of Veterinary Science (UVAS), located in Lahore, Pakistan, for comprehensive hematological analysis. The analysis included the determination of various parameters such as red blood cell count (RBC), white blood cell count (WBC), monocytes, neutrophils, lymphocytes, eosinophils, hemoglobin (Hb), hematocrit (HCT), platelet count, mean corpuscular volume (MCV), mean corpuscular hemoglobin concentration (MCHC), and mean corpuscular hemoglobin (MCH).

### 2.8 Blood biochemical analysis

The present study involved the collection of blood samples from ten zebrafish in each group. A complete cell count was performed to identify any irregularities in the cell numbers of all types. However, it was deemed unnecessary to examine other parameters, as the amount of blood collected was minimal, and only a limited number of zebrafish were sacrificed.

### 2.9 Dissection of Zebrafish

Five Zebrafish were selected from each of the seven aquariums for dissection. Prior to the procedure, the Zebrafish were anesthetized with 0.2 percent Tricaine and euthanized by placing them in ice water for 10–15 minutes. The fish were then placed on a dissection mat and gently patted dry with a paper towel. During the dissection process, the Zebrafish’s paired pectoral and pelvic fins, as well as its single dorsal, caudal, and anal fin, were identified and noted. The Zebrafish was then secured to the cutting mat by tethering through the fleshy portion of the tail and the ventral portion of the eye socket. The skin on the belly, just in front of the anal fin, was plucked. Beginning at the anal fin and moving towards the operculum, the skin and underlying muscle along the belly were trimmed. The operculum, pectoral fin, and pectoral girdle were then removed. Next, the skin and underlying muscle were cut along the side of the fish, extending down to the anal fin. Finally, the skin and underlying muscle were carefully removed from the side of the fish, allowing for the observation of the internal organs. The organs were then separated and identified individually. These organs included the liver, heart, brain, intestine, swim bladder, and ovaries or testes, depending on the sex of the Zebrafish.

### 2.10 Histopathology

The histological investigation of the following essential organ was conducted to evaluate the structural alterations in tissues i.e Sex organs, Brain, Liver, Heart, Gills, and Intestine. Hematoxylin and Eosin (H & E) staining was done and histopathological analysis was done by a qualified veterinary pathologist at UVAS.

### 2.11 Fish embryo acute toxicity test (FET)

Fertilized zebrafish eggs were subjected to a 96-hour exposure to recombinant algae containing cry11Ba protein at 2mL, with observations made every 24 hours. The test solution was replaced daily to ensure accurate results. The experiment involved three concentrations of the chemical under test, administered to a 24-well plate containing one embryo per well. The control used was vector pSRSap1. The observations were made every 24 hours to assess signs of lethality or abnormality, and acute toxicity was identified if there was a favorable impact in any of the recorded observations. The zebrafish eggs and larvae did not display any negative impacts from the experiment, and every embryo developed into a normal larva without any abnormal development. Similar tests have been conducted previously by Rebecca et al. [35–37].

### 2.12 Mosquito larvicidal bioassay

Mosquito larvicidal bioassays were performed to validate the western blot findings. Only the accumulation of cry11Ba protein was assessed, followed by an evaluation of mosquito larvicidal activity to determine the expression of cry proteins. cry11Ba algal cells were administered to 2nd instar *Aedes aegypti* larvae in three different concentrations. We utilized pSRSap1 as negative controls.

### 2.13 Statistical analysis

The statistical analysis was conducted to compare the treatment groups consuming different feed concentrations. The statistical evaluation of food consumption, body weight, and body length was performed using one-way ANOVA. If the data was found to be significant, a t-test may also have been conducted. All statistical analyses were performed using GraphPad Prism version 8.0.1 (Inc., San Diego, CA). The significance level was set at p < 0.05.

## 3. Results

The experimental period witnessed the maintenance of optimal health and normal behavior in all the Zebrafish. It is noteworthy that no fatalities, near-fatal incidents, allergic reactions, or aberrations in the usual behavioral patterns were observed in any of the groups.

### 3.1 PCR for the validation of gene of interest in test substance

**Fig 1:**
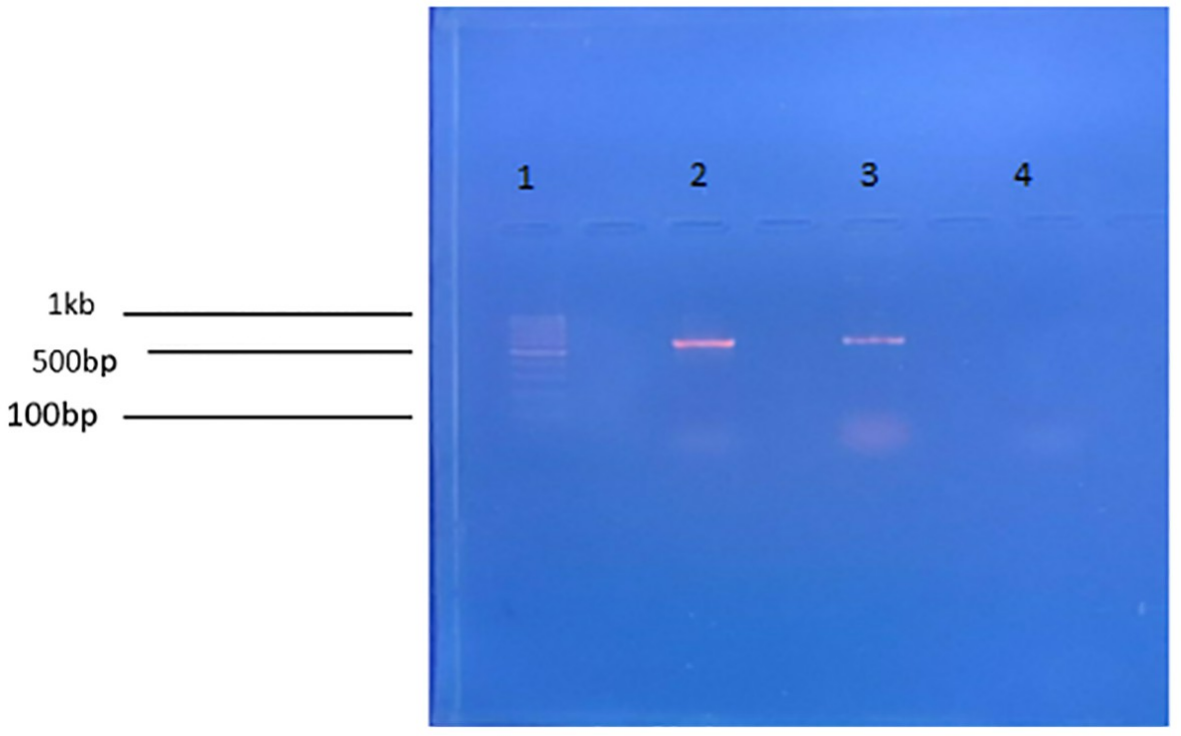
PCR for the validation of cry11Ba gene. Column 1: 100bp (Thermoscientific ladder), Column 2: 616bp PCR product of cry11Ba gene. Column 3: +ve control, Column 4: -ve control.

The presence of the cry11Ba gene in the substance under test was confirmed by the detection of a 616bp band, as shown in Fig 1. To ensure the validity of the result, a 100 bp ladder, a positive control containing cloned plasmid DNA, and a negative control were included and analyzed alongside the substance under test. This was done to establish the reliability and accuracy of the findings, which were found to be consistent with the results obtained from the positive control.

### 3.2 Clinical pathology

During the course of the experiment, there were no reports of any adverse effects on the Zebrafish, including death, sickness, or harmful consequences. Furthermore, no observable changes were noted in their skin, eyes, or general appearance. Upon conducting an evaluation of the functional parameters, it was determined that there were no unusual responses exhibited by any of the Zebrafish groups.

### 3.3 Body weight

The body weight before and after the experiment was measured. The statistical analysis revealed no significant differences (p>0.05) among all the groups.

### 3.4 Body length

The body length before and after the experiment was measured. The statistical analysis revealed no significant differences (p>0.05) among the groups.

### 3.5 Blood biochemical analysis

There were no indications of toxicological effects in the blood samples collected from all groups fed with different diet combinations.

### 3.6 Hematology

Hematology results are discussed below. No significant differences were found among all the groups as indicated in Table 1.

**Table 1:** Hematology results of Zebrafish.

Test Performed	Group1	Group2	Group3	Group4	Group5	Group6	Group7
Hemoglobin (Hb)	7 g/dl	7.4 g/dl	6.9 g/dl	8.2 g/dl	7.6 g/dl	8 g/dl	7.8 g/dl
WBCs	5.3x10^^3^/μL	5.4x10^^3^/μL	5.4 x10^^3^/μL	5.3 x10^^3^/μL	5.2 x10^^3^/μL	5.3 x10^^3^/μL	5.4 x10^^3^/μL
RBCs	3.3x10^^6^/μL	3 x10^^6^/μL	3.2x10^^6^/μL	3.2 x10^^6^/μL	3.1 x10^^6^/μL	3.02 x10^^6^/μL	3 x10^^6^/μL
HCT	28%	32%	27%	29%	31%	29%	30%
MCV	89fL	96.6fL	92.8fL	101fL	96fL	85fL	100fL
MCH	15.2pg	15.5pg	15.4pg	15.5pg	15.2pg	15.5pg	15.3pg
Platelet count	32 x10^^3^/μL	28 x10^^3^/μL	25 x10^^3^/μL	30 x10^^3^/μL	28 x10^^3^/μL	20 x10^^3^/μL	25 x10^^3^/μL
Neutrophils	49%	49%	50%	49%	51%	49%	48%
Lymphocytes	32%	29%	33%	32%	34%	31%	29%
Monocytes	06%	08%	08%	08%	06%	08%	08%
Eosinophils	0.7%	01%	0.8%	01%	0.9%	01%	0.8%

### 3.7 Morphology of organs

**Fig 2:**
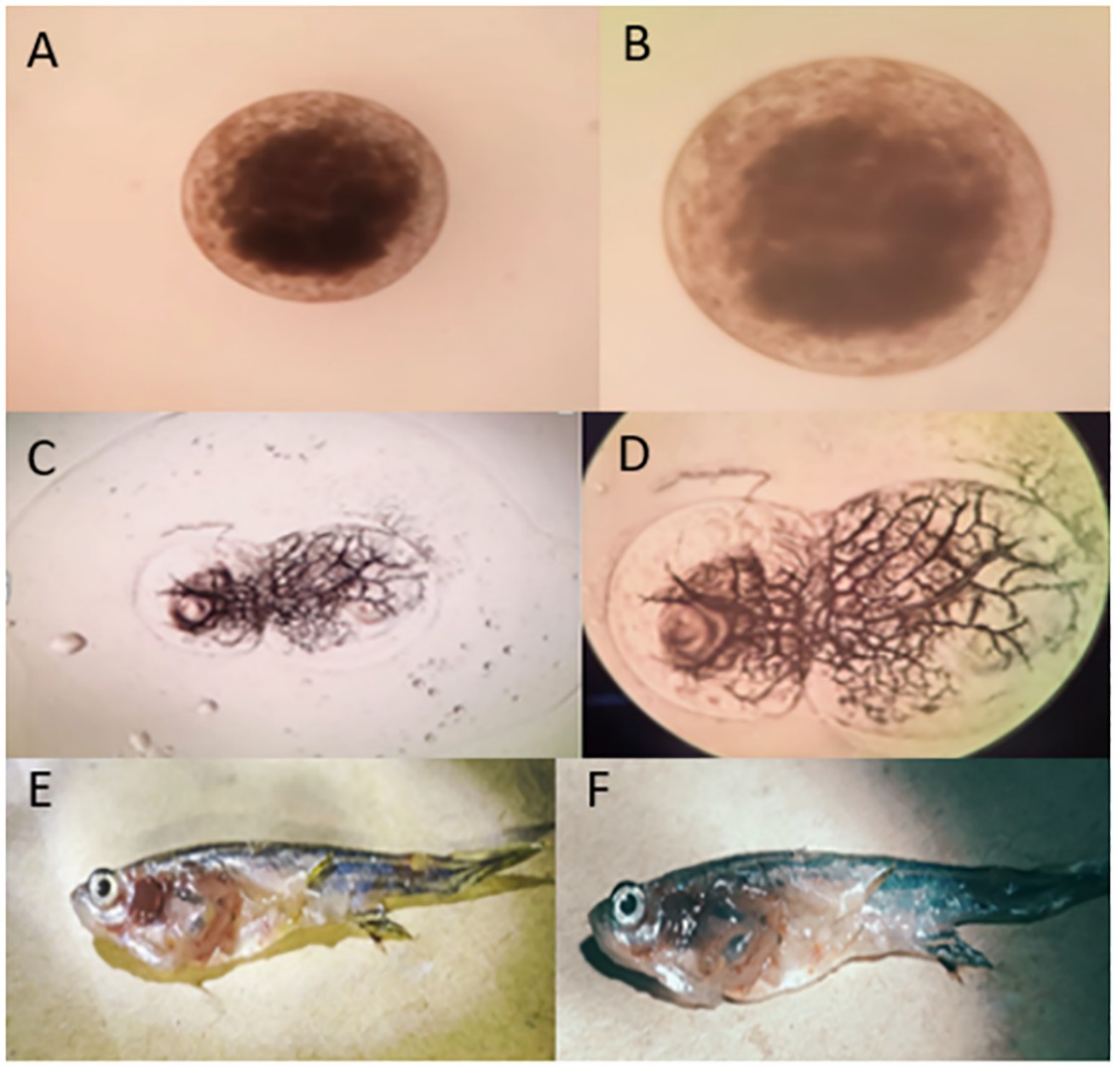
(A, B)Fertilized Zebrafish eggs before hatching (C, D) Zebrafish embryo at initial developmental stages (E, F) The morphology of Zebrafish organs and skin after dissection.

No anomalies, such as the presence of lesions, alterations in organ color, texture, or organ necrosis, were observed in any of the zebrafish across all the experimental groups (Fig 2).

### 3.8 Histopathology

**Fig 3:**
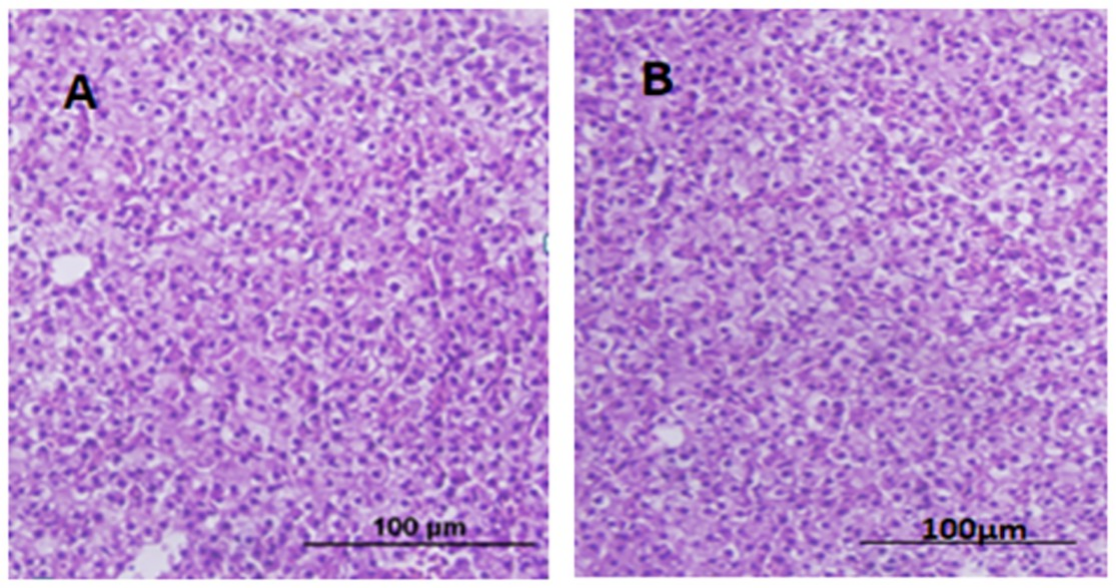
Liver tissues with H & E staining. (A) represents the test group, (B) represents the control group. Scale bar = 100μm.

**Fig 4:**
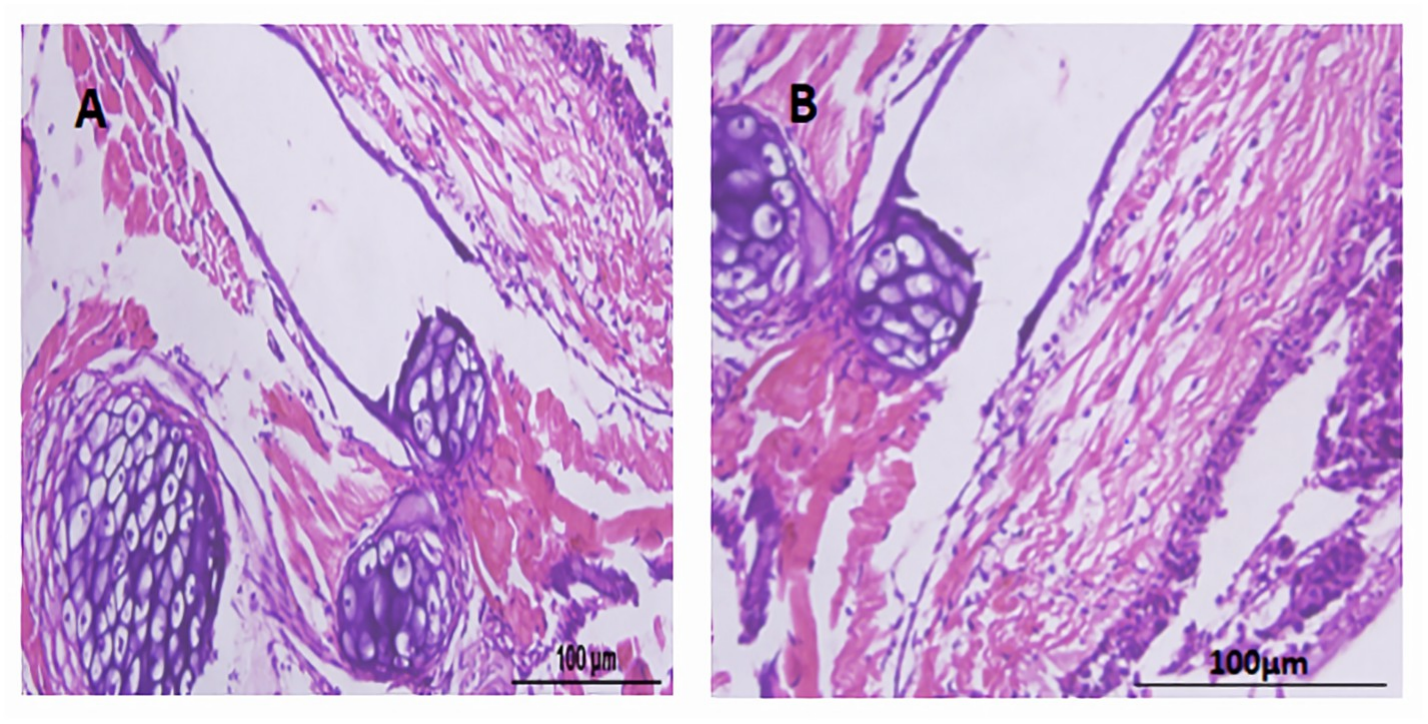
Ovarian tissues with H & E staining. (A) represents the test group, (B) represents the control group. Scale bar = 100μm.

**Fig 5:**
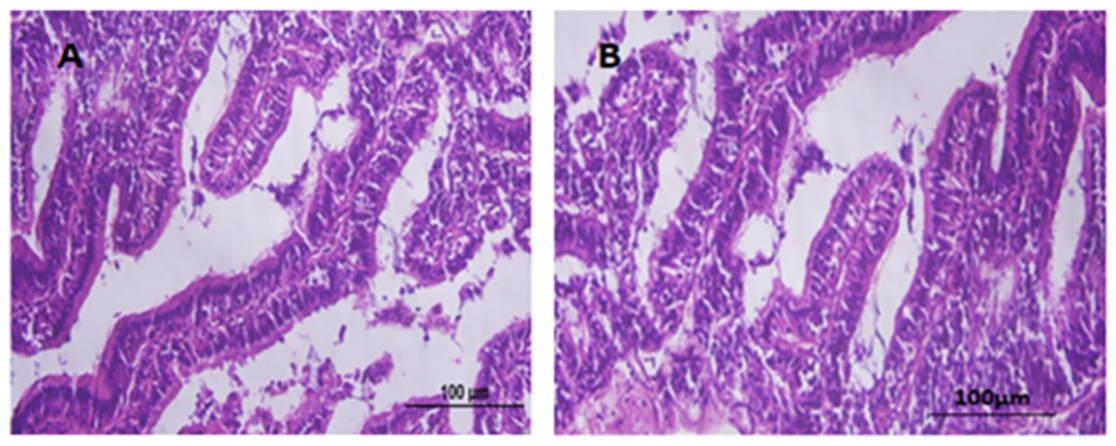
Intestinal tissues with H & E staining. (A) represents the test group, (B) represents the control group. Scale bar = 100μm.

The slides showcasing the crucial organs of fish, comprising of the liver, intestine, heart, gills, brain, and sex organs, were ready and stained with H&E. These slides were then assessed under a compound microscope at magnifications of 100X, 200X, and 400X. Upon a comprehensive evaluation and consultation from experts, it was concluded that no conspicuous disparities were observed in the microscopic examination of vital organs across all the groups, as depicted in Figs 3–5.

### 3.9 Molecular analysis

#### 3.9.1 PCR to check for integration of cry11Ba in Zebrafish tissue

No bands were detected in the DNA samples from the test groups as indicated in Fig 7.

#### 3.9.2 Western blot

Western blotting was done to confirm that there is no gene integration in the zebrafish tissues, which further ensures the safety of this transgenic gene for non-target organisms.

### 3.10 Mosquito larvicidal bioassay

It was discovered that all the mosquito larvae that were fed either live or dried cry11Ba algal cells were found to be deceased after a period of 72 hours. This finding demonstrated that the algae possessed a larvicidal active gene that had proven lethal to the larvae.

## 4. Discussion

The present study was conducted to assess the toxic effects of a mosquito larvicidal genetically engineered microalgae (*C*. *reinhardtii*) on *Danio rerio* (zebrafish), in which transgenic algae mixed with commercially available feed were delivered orally as feed to zebrafish in aquarium water. This feeding mode was chosen as it is advantageous because algae can grow naturally in water bodies and is self-sustainable [38–40]. Furthermore, as algae grow in water, they may live in the medium for longer periods of time. Finally, this transgenic algae is meant to be used as a mosquito larvicidal spray in water bodies where off-target species can also feed on it. Therefore, it is most appropriate to test biosafety in animals (zebrafish) in water. Murbach et al., [32], Fields et al., [41], and Kumar NA et al., [38] have also conducted biosafety studies of *C*. *reinhardtii* by oral feeding on test animals.

Zebrafish were used in this study for a 90-day feeding trial with GM algae (*Chlamydomonas reinhardtii*) at concentrations of 1x10^5^cells/mL, 2x10^5^cells/mL, and 5x10^4^cells/mL. After thorough toxicity analysis of the different groups (transgenic, non-transgenic, and control groups) of zebrafish, no mortality was observed. Weight and length before and after the experiment were similar and showed no significant differences among the seven groups. Furthermore, the haematological analysis did not show any abnormal effects, as shown in Table 1, which shows the Complete Blood Count (CBC) results of zebrafish from all the experimental groups and control group. The results of our study correspond to those of Murtha et al., who reported similar data [42, 43].

In the current study, no change was observed in body weight and body length before and after feeding the transgenic algae shown in S1 Table. The morphology of various zebrafish organs was observed to be normal after the experimental period (Fig 2). Vital organ (Liver, Sex organs, intestine) tissues were used for histopathological analysis (Figs 3–5). The microscopic appearance of zebrafish tissues showed no significant differences among all the groups which also aligns with the previous work of Pack et al. [44–46]. Organ weight, morphology, and histopathological examination revealed no significant changes. AL Menke et al., in their study showed the normal histology of adult Zebrafish [47]. The results of this study correlate with those of Grisolia et al. study, as they did not report any abnormalities or any visible adverse effects on zebrafish after exposure to the test compound [48]. This study showed normal cell and tissue morphology in zebrafish.

Allergenicity, body weight, and mortality are sensitive predictors of toxicity in biosafety investigations [49]. All Zebrafish were evaluated once a day for mortality, morbidity, and allergenicity. Individual body weights were determined at the start and end of the experiment. Clinical observations revealed no evidence of allergy or toxicity.

**Fig 6:**
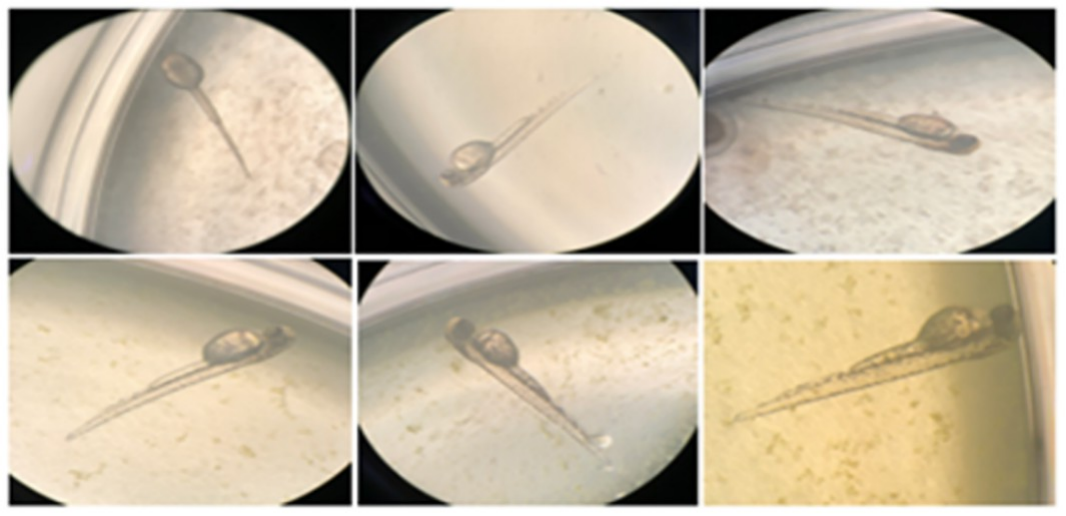
Zebrafish larvae at 32hrs, 40hrs, 56hrs, 64hrs, 72hrs and 96hrs respectively.

**Fig 7:**
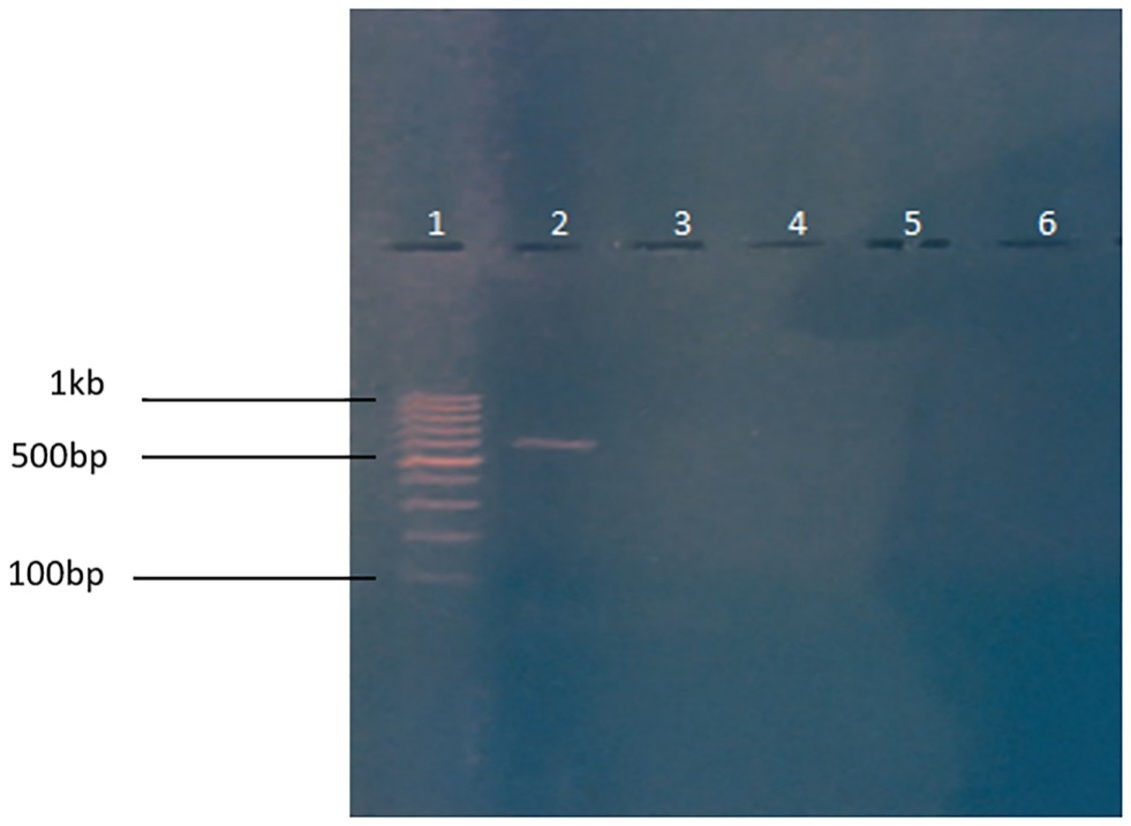
PCR to confirm there is no test gene integration in Zebrafish tissues. Column 1: Shows 100bp Ladder (Thermoscientific). Column 2: Shows +ve control cry11Ba, Column 3: Shows -ve control wild type, Column 4: Shows Zebrafish intestine DNA, Column 5: Shows Zebrafish liver DNA, Column 6: Shows Zebrafish heart DNA.

**Fig 8:**
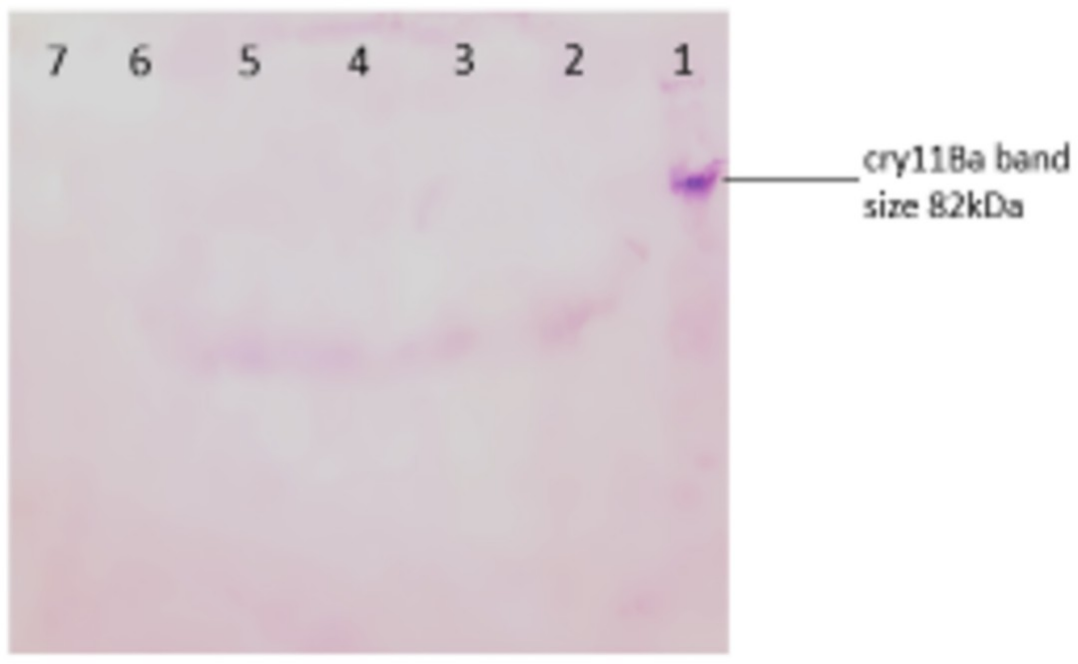
Western blot for the confirmation of no gene integration in Zebrafish tissues. Lane 1 shows positive control cry11Ba, Lane 2–4 are from experimental groups. Lane 5–7 are from control groups.

Vieira et al. (2021) observed similar results in their study. Insecticidal proteins from Bacillus thuringiensis (Bt), Cry1C, Cry1F, and Cry1Ab, were expressed in transgenic plants. It is crucial to assess the effects of these proteins on aquatic organisms, given that they enter aquatic settings [50]. These proteins did not have any negative effects on the measured concentrations during the early life stages of zebrafish. Our study showed similar results as described (Fig 6), which showed normal development of zebrafish embryos for 4 days during the experiment in which the larvae were treated with the transgenic algae having the mosquito larvicidal gene. This test also proved to be strong evidence in support of this study, as the transgenic algae did not cause any harm to zebrafish embryos and they developed into larvae without showing any abnormality. Furthermore, no bands were detected in the DNA samples from the test groups which verified that the gene was not incorporated into the organism after its intake (Fig 7). Mosquito larvicidal bioassay shown in S2 Fig was carried out to confirm the western blot results. Mosquito larvae died when they were treated with transgenic algae with mosquito larvicidal activity (Fig 8).

In a previous study by Kwon et al. (2019), it was reported that orally administered vaccines based on algae showed potential benefits. When tested in zebrafish, no negative or toxic effects were observed [39]. Therefore, all results fell within the normal range, and no biologically significant differences were found among the experimental groups. These findings were consistent with those of previous studies by RM Darwish et al., [51], EC Oliveria-Filho et al., [52] and MN Haque et al. [53], which also demonstrated no adverse effects on fish fed with genetically modified (GM) algae. It is worth noting that there is a well-established history of the safe consumption of Bt proteins, such as Cryl1Ba. The mammalian intestine remains unaffected by these proteins because of the lack of corresponding receptors for Bt and the presence of an alkaline pH [54, 55].

## 5. Conclusion

In conclusion, the results of the three-month experiment conducted on zebrafish exposed to genetically modified algae (*Chlamydomonas reinhardtii*) containing cry11Ba indicate that there were no detrimental consequences on their overall health. This suggests that unlike conventional pesticide sprays, which have harmful effects on living organisms, genetically modified algae can be used to ensure the safety of living organisms by eliminating target organisms, such as mosquitoes, at the larval stage. The outcomes of this study may contribute to the successful deployment and commercial release of genetically modified algae without any adverse effects.

## Supporting information

S1 Fig Experimental setup. (PDF)

S2 Fig Bioassay of transgenic algae. The dead mosquito larvae after exposure to transgenic algae and non-transgenic algae.
(PDF)

S1 Table Shows weight of Zebrafish. (PDF)

S1 Graphical abstract (DOCX)
