# Supplementary figures and images for: An oral toxicity assessment of a mosquito larvicidal transgenic algae (Chlamydomonas reinhardtii) using adult Zebrafish and its embryos
Source: PLoS One. 2024 Jun 13;19(6):e0303352. doi: 10.1371/journal.pone.0303352 (PMC11175461; doi:10.1371/journal.pone.0303352)

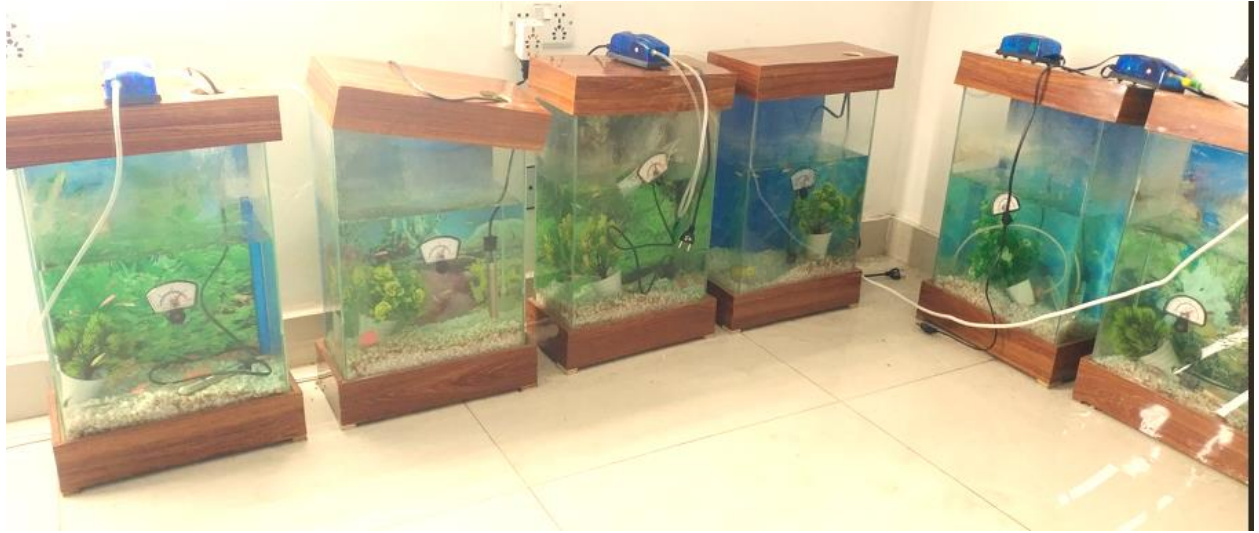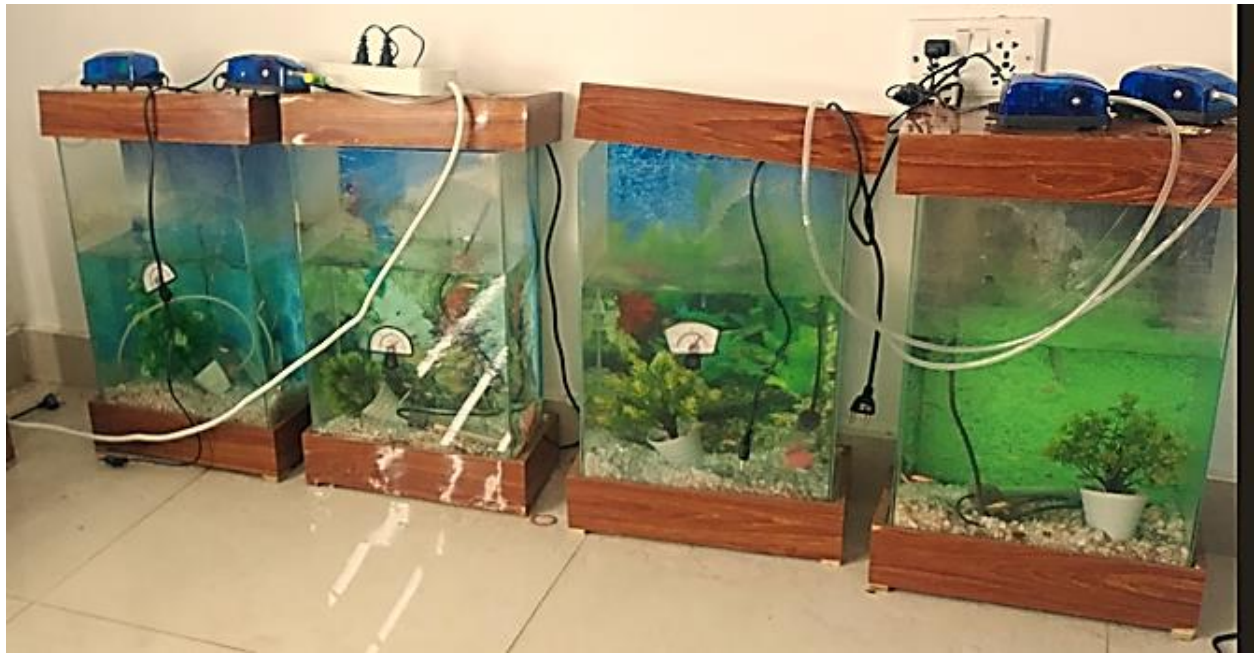

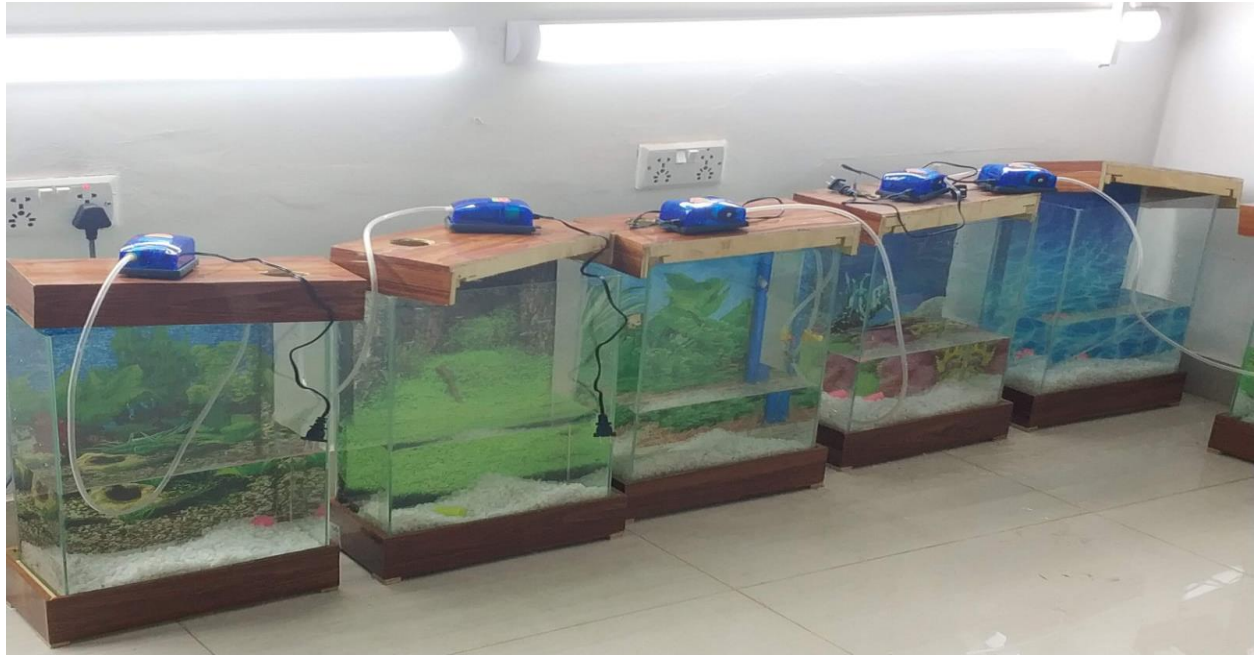

Supplement: S1 Fig — (PDF) [file pone.0303352.s001.pdf]

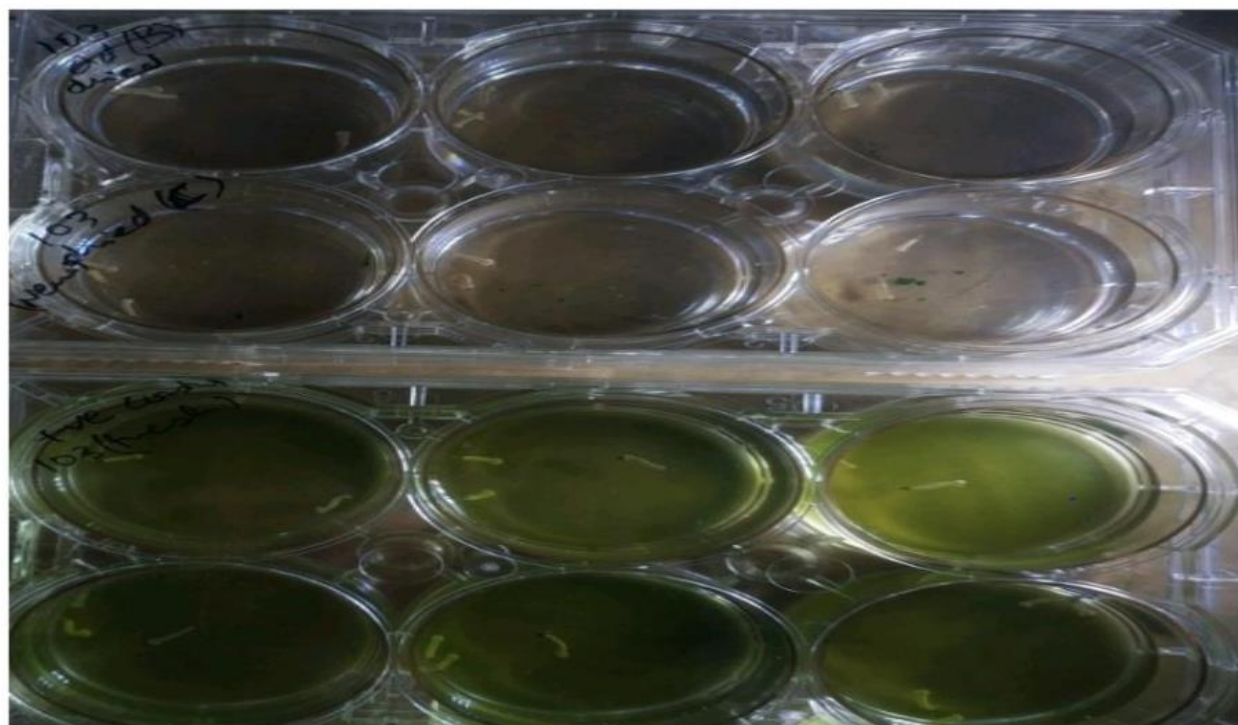

Supplement: S2 Fig — The dead mosquito larvae after exposure to transgenic algae and non-transgenic algae. (PDF) [file pone.0303352.s002.pdf]

**Graphical Abstract**


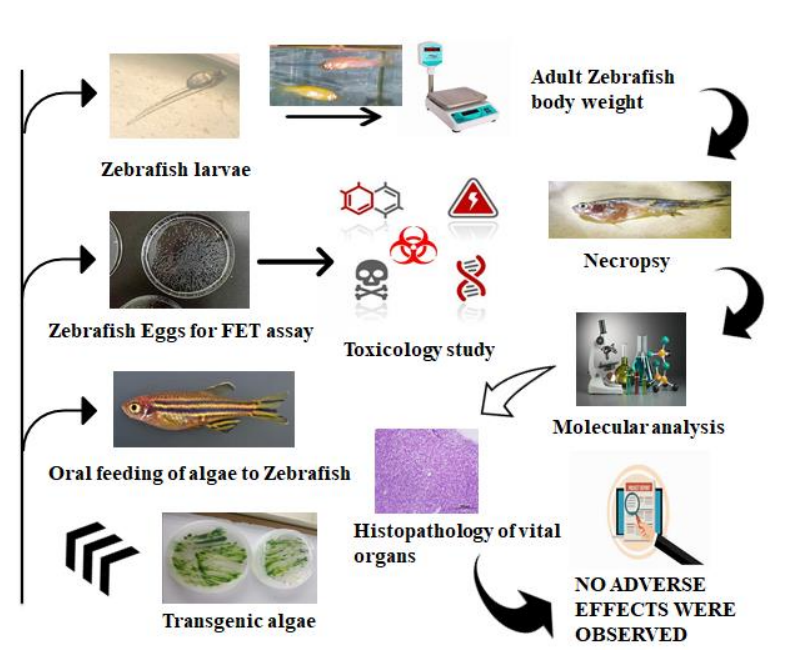

Supplement: S1 Graphical abstract — (DOCX) [file pone.0303352.s004.docx]
