# Supplementary material for: An oral toxicity assessment of a mosquito larvicidal transgenic algae (Chlamydomonas reinhardtii) using adult Zebrafish and its embryos
Source: PLoS One. 2024 Jun 13;19(6):e0303352. doi: 10.1371/journal.pone.0303352 (PMC11175461; doi:10.1371/journal.pone.0303352)

Weeks	Test Group	Control Group
2	0.3	0.278
4	0.216	0.212
6	0.348	0.342
8	0.36	0.358
10	0.318	0.266
12	0.434	0.284
14	0.322	0.266

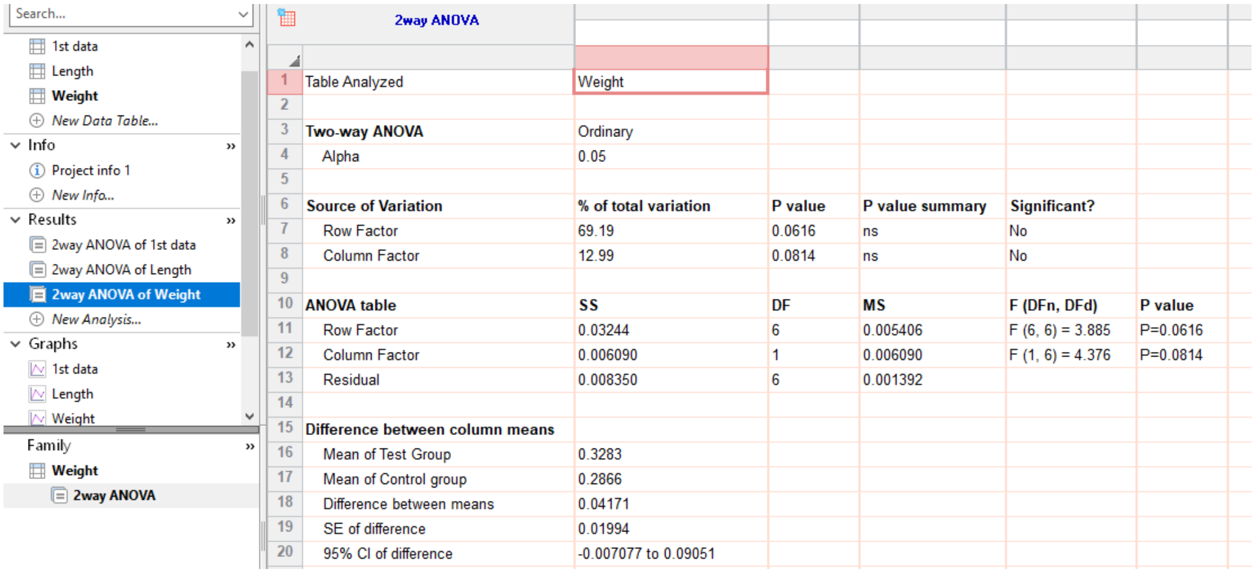

1	Table Analyzed	Weight				
2						
3	Two-way ANOVA	Ordinary				
4	Alpha	0.05				
5						
6	Source of Variation	% of total variation	P value	P value summary	Significant?	
7	Row Factor	69.19	0.0616	ns	No	
8	Column Factor	12.99	0.0814	ns	No	
9						
10	ANOVA table	SS	DF	MS	F (DFn, DFd)	P value
11	Row Factor	0.03244	6	0.005406	F (6, 6) = 3.885	P=0.0616
12	Column Factor	0.006090	1	0.006090	F (1, 6) = 4.376	P=0.0814
13	Residual	0.008350	6	0.001392		
14						
15	Difference between column means					
16	Mean of Test Group	0.3283				
17	Mean of Control group	0.2866				
18	Difference between means	0.04171				
19	SE of difference	0.01994				
20	95% CI of difference	-0.007077 to 0.09051				

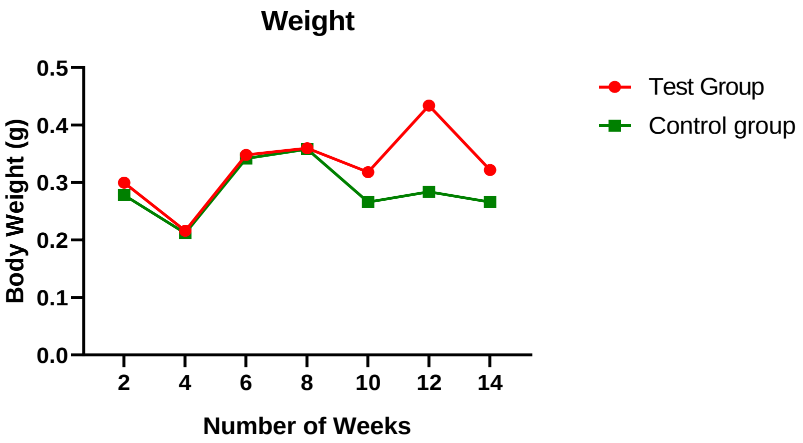

Supplement: S1 Table — (PDF) [file pone.0303352.s003.pdf]
